# AIR: A batch-oriented web program package for construction of supermatrices ready for phylogenomic analyses

**DOI:** 10.1186/1471-2105-10-357

**Published:** 2009-10-28

**Authors:** Surendra Kumar, Åsmund Skjæveland, Russell JS Orr, Pål Enger, Torgeir Ruden, Bjørn-Helge Mevik, Fabien Burki, Andreas Botnen, Kamran Shalchian-Tabrizi

**Affiliations:** 1 Microbial Evolution Research Group (MERG), Department of Biology, University of Oslo, Norway; 2 Centre of Information Technology, University of Oslo, Norway; 3 Department of Botany, University of British Columbia, Vancouver, British Columbia, Canada

## Abstract

**Background:**

Large multigene sequence alignments have over recent years been increasingly employed for phylogenomic reconstruction of the eukaryote tree of life. Such supermatrices of sequence data are preferred over single gene alignments as they contain vastly more information about ancient sequence characteristics, and are thus more suitable for resolving deeply diverging relationships. However, as alignments are expanded, increasingly numbers of sites with misleading phylogenetic information are also added. Therefore, a major goal in phylogenomic analyses is to maximize the ratio of information to noise; this can be achieved by the reduction of fast evolving sites.

**Results:**

Here we present a batch-oriented web-based program package, named AIR that allows 1) transformation of several single genes to one multigene alignment, 2) identification of evolutionary rates in multigene alignments and 3) removal of fast evolving sites. These three processes can be done with the programs AIR-**A**ppender, AIR-**I**dentifier, and AIR-**R**emover (AIR), which can be used independently or in a semi-automated pipeline. AIR produces user-friendly output files with filtered and non-filtered alignments where residues are colored according to their evolutionary rates. Other bioinformatics applications linked to the AIR package are available at the Bioportal , University of Oslo; together these greatly improve the flexibility, efficiency and quality of phylogenomic analyses.

**Conclusion:**

The AIR program package allows for efficient creation of multigene alignments and better assessment of evolutionary rates in sequence alignments. Removing fast evolving sites with the AIR programs has been employed in several recent phylogenomic analyses resulting in improved phylogenetic resolution and increased statistical support for branching patterns among the early diverging eukaryotes.

## Background

A well-resolved phylogenetic tree demonstrating the relationships between species is one of the most important goals in evolutionary biology, and the fundament for comparative studies in many fields in life science. Multiple gene sequence data is increasingly being used to resolve phylogenetic relationships, and frequently more than 50 genes are being inferred to address key questions about the early evolution of eukaryotes [1-8]. Recent studies have for instance shown support for the grouping of known eukaryotes into a handful of supergroups [2,5,9-15]. The main reason for constructing multigene data instead of using single gene data in phylogenetic reconstruction is to collect enough information to improve the phylogenetic signal [9,16]. Accordingly, as the number of genes increases, the tendency is that phylogenetic relationships are better resolved and receive higher statistical support [2,5,16-18]. However, simply adding genes to an alignment to increase statistical support does not necessarily lead to more accurate results; inconsistencies in datasets may adversely lead to higher support for an incorrect topology. Reducing such stochastic errors is an important step in improving the phylogenetic resolution of the sequence data [16,19-21]. Consistency in the data may be improved by the removal of the fastest evolving sites; as such sites may have over-representation of substitution saturation causing homoplasies [22,23]. However, so far only a few bioinformatics program has been reported that allows for the concatenation of multiple single gene alignment files, identification of fast evolving sites and removal of fast evolving sites in accordance with the users needs.

**Figure 1:**
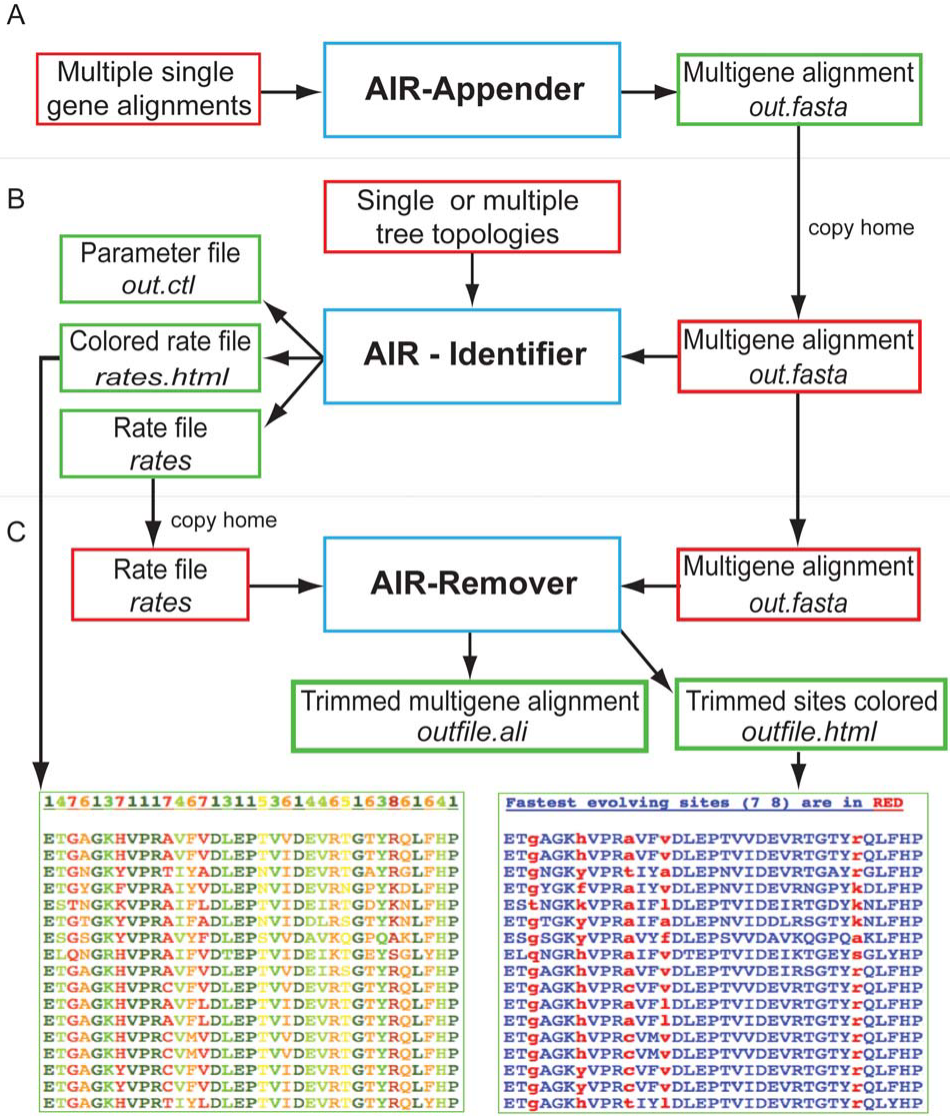
**Overview of AIR-package**. Overview of the functionalities and programs in the AIR-package installed on the Bioportal: The colored boxes depict input files (red), output files (green), and the AIR programs (Blue). Texts in *Italics *depict the filename and respective extension of output files of AIR programs. A) AIR-Appender uses several single gene alignments for construction of a multigene alignment. B) AIR-Identifier uses the output file from AIR-Appender and file containing one or more phylogenetic trees for calculating site rates and rate categories. C. AIR-Remover deletes fast evolving sites according to settings defined by the user. The output files from each of the AIR programs can be used in subsequent analysis by copying the files from the work directory to project folder on the Bioportal using the *copy home *function. Five main output files are produced by AIR. In which two are graphical html files with information about site rates and fast evolving sites (*rates.html*), and sites removed from the alignment (*outfile.html*). File 'rates.html' shows the rate categories as different colors (up to 8 categories), while '*outfile.html*' shows the removed sites in red color (e.g. category 7 and 8 removed are shown in red), and rest sites in blue. Files namely '*rates*' and '*out.ctl*' are produced by PAML programs, which are implemented in AIR-Identifier. While '*outfile.ali*' is the multigene alignment with fast evolving sites removed.

Here we present a bioinformatics package, named AIR that combines all these possibilities. AIR is divided into three applications: AIR-**A**ppender, AIR-**I**dentifier and AIR-**R**emover (Figure 1). AIR-Appender performs separate processing of data by appending single gene alignment files to a multi-gene alignment. AIR-Identifier identifies fast evolving sites by calculating site-rates, and AIR-Remover removes fast evolving sites from an alignment. The AIR programs are interlinked with other applications useful in the field of phylogenomics (i.e., multi-gene BLAST, contig assembly of Sanger and 454 sequences, alignment and phylogeny) through the Bioportal at the University of Oslo.

## Implementation

The AIR package is implemented on the Bioportal at the University of Oslo. The Bioportal is a web-based bioinformatics service freely available to academic users at the following URL: . The Bioportal uses SQL for maintaining information about users, files, databases, and jobs. The Bioportal resources are deployed on Linux with Apache HTTP server 2.2. The critical scripts to maintain the Bioportal, e.g. cron jobs scripts and post-processing scripts, are written in Perl v5.8, and python 2.3. The web-interface for all available applications on Bioportal is written in PHP 4.3.

Each user of the Bioportal has access to several file directories and file administration functions. All files used as input for analyses are stored in project folders defined by the users. Once the user has created a project folder they can upload data-files into its respective project folders. The user can then use the web interface created for each application on Bioportal to select their files, applications (here for example AIR-Appender, AIR-Identifier, or AIR-Remover) and parameter settings. For each analysis a working folder is created in the working directory 'job admin'. A 'copy home' function in the 'job admin' can be used to transfer files from working directories to project folders; hence result files from one process can be used as input files in subsequent analyses, and to link different applications in a semi-automated pipeline. For instance, alignments made by MAFFT [24] can be used for phylogenetic analyses by one of the available phylogenetic programs e.g. RAxML, Treefinder or MrBayes [25-27]. The Bioportal tutorial is available at the Bioportal website.

All successfully submitted Bioportal jobs are run in the background, the execution time of each process varies dependent on the file size and the nature of the selected applications. To keep track of the status of submitted jobs a manager module has been developed on the Bioportal; this updates the users about the current status of all jobs. Upon completion the results are returned to the respective working directory where files can then be downloaded in a compressed 'zip' format.

Currently the Bioportal is the largest high performance-computing environment in Norway. The available computer resources are 320 dedicated cores on the TITAN cluster at the University of Oslo. In addition, the Bioportal has access to all free or idle TITAN cores if needed (4000 at present). The TITAN cluster has LINUX nodes with 16 gigabytes of memory and 2× quadcore CPUs or 2× dual-core CPUs.

## Results

### Appending single gene alignments

AIR-Appender merges multiple single gene alignment files into one major multigene alignment; the program looks for species with identical names and subsequently merges these. If any of the single gene alignments are lacking taxa in relation to one another, the program will automatically replace the missing data with question marks '?'. The junction between genes will be marked with double hyphen for easy identification of the sequence borders. The resulting output of AIR-Appender is a single FASTA and PAML formatted file containing the multiple gene alignment (*out.fasta *in Figure 1); this can be used for downstream processing with AIR-Identifier (or other programs available on the Bioportal) or downloaded to a local computer as a compressed zip file.

### Identifying site rate

**Figure 2:**
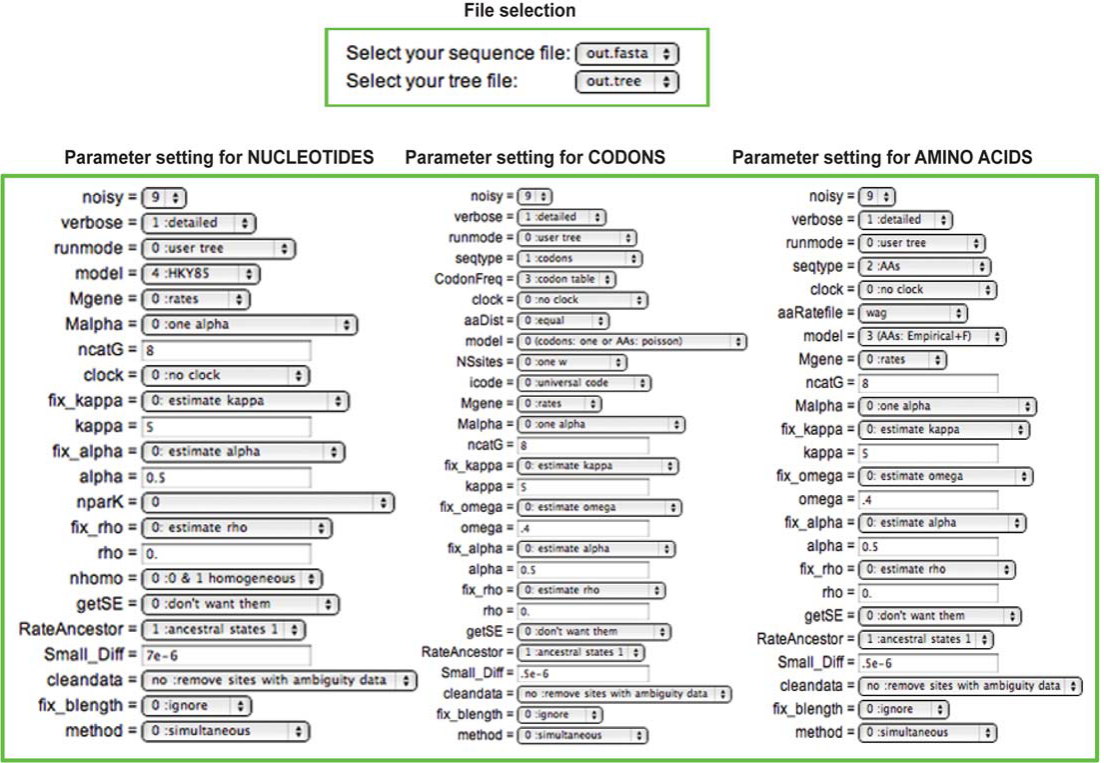
**AIR-Identifier Web-Interface**. AIR-Identifier web-interface on the Bioportal, where the user can select input files (i.e. sequence alignments and tree file containing phylogenetic trees) and parameters for three types of data; i.e. nucleotides, codons, and amino acids. The sequence files can be in FASTA or PAML format, while single or multiple trees in the tree file must be in Newick format and supplied in a single file.

After the user has made the multi-gene sequence file, site-rates (i.e. posterior mean values) can then be identified for nucleotides, codons and amino acids sequences with the program AIR-Identifier. AIR-Identifier applies the PAML programs codeml (for codon and amino acid sequences) and baseml (for nucleotide sequences) [28,29]. The control file (*out.ctl *in Figure 1) is critical as it is here that the user defines a set of parameters to be used for estimation of site rates by codeml or baseml. These programs are usually only available via the command line, and thus setting parameters for a successful run can be a cumbersome task. We have therefore developed AIR-Identifier as a user-friendly web interface for the PAML programs; here the users can define the parameters and their respective values (Figure 2). For instance, the evolutionary model for calculation of site-rates, and the number of rate categories (normally 8 categories) for the analysis can be defined. Users still have an option to use their own control file that can be uploaded to the Bioportal.

Two types of files are used to calculate the site rates: 1) a multigene alignment in FASTA format with file extension '.fasta' or PAML format, and 2) a corresponding file containing a phylogenetic tree. The tree file should be generated with a suitable phylogenetic programs; the codeml and baseml programs are not recommended to reconstruct trees (see the PAML manual [30]). The tree topologies accepted are typically specified using the parenthesis notation such as the Newick tree format [31]. It should be noted that some widely used programs such as PAUP or MacClade [32,33] can produce tree files with limited compatibility, whereas other programs such as PHYLOBAYES v. 2.3 [34] or RAxML-VI-HPC [27] generate output files that are ready to use. Trees with or without branch length are accepted by AIR-Identifier.

It can often be difficult to decide which phylogeny should be used for estimating rates, especially when a dataset gives differing trees from different evolutionary models, parameters and tree searching algorithms. It has also been proposed that the selection of phylogeny can have a major impact on rate estimation [21]. For this reason we have constructed the AIR-Identifier to calculate site rates and rate categories from multiple phylogenetic trees.

The AIR-Identifier program produces two output files: 1) A rate file, which contains information about the evolutionary rate (rate category) for each site in the alignment (*rates *in Figure 1); 2) A html file (i.e. *rates.html *in Figure 1) that visually presents information about the rate pattern in the alignment and which allow the users to easily evaluate the importance of the various rate categories and the dispersal of the site rates along the alignment before sites are removed; the file also includes an graphical overview of the alignment where different rate categories have been color-coded.

### Removing fast evolving sites

**Figure 3:**
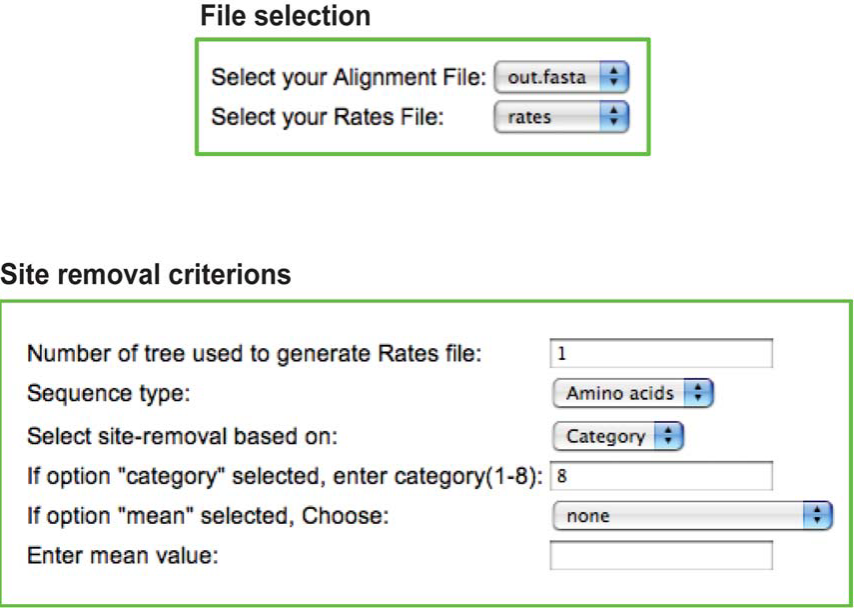
**AIR-Remover Web-Interface**. AIR-Identifier uses *rates *generated with AIR-Identifier (Figure 1) and the corresponding multigene alignment in PAML format. Sites can be removed on the basis of site rates or rate categories.

AIR-Remover is developed for the removal of fast evolving sites. The sites can be removed based on either site-rate or rate-category. The AIR-remover uses the alignment file and respective *rates *file obtained as output from AIR-Identifier. The users can then decide which of the rates and categories of fastest evolving sites should be removed. Multiple categories can be removed by using comma-separated numbers. The users can also remove sites that correspond to a fraction of the fastest evolving sites by defining a percentage of the total rate distribution; it is possible to remove e.g. the 5% fastest evolving sites (Figure 3). The AIR-Remover output files produces a main result file containing the ready to use alignment file (*outfile.ali *in Figure 1) and an html file (*outfile.html *in Figure 1) that enables the users to visualize the removed sites colored in red within their alignment.

## Discussion and conclusion

The AIR package has been extensively used in recently published phylogenomic studies of deeply diverging eukaryote lineages [2,18]. In the study of Burki et al., 2008, a global eukaryote phylogeny was reconstructed from a dataset of 135 genes and 65 taxa, resulting in 73% bootstrap support for a single "megagroup" comprising nearly all photosynthetic lineages (including the supergroups Plantae, chromalveoalates and Rhizaria). When the fast evolving sites were identified and removed from the alignment with AIR, the same topology was recovered but with a substantially increased bootstrap support (97%) for the observed relationship. In the study of Minge et al. 2008, the evolutionary position of an enigmatic lineage named *Breviata *was in question using 78 genes and 38 taxa. The lineage was placed with strong bootstrap support as sister to the supergroup Amoebozoa, however statistical testing i.e. AU-test [35] of alternative placements in the eukaryote tree could not reject a sister relationship to another supergroup, the Excavata. Once fast evolving sites were removed using AIR the AU test could reject an affinity to the Excavata and additionally placed *Breviata *with the Amoebozoa with higher bootstrap support. Interestingly, the removal of additional fast evolving sites (altogether the 3 fastest rate categories) reduced the bootstrap support for the monophyly of *Breviata *and Amoebozoa, thus suggesting that the removal of too many categories or sites can reduce relevant phylogenetic information in the data. It demonstrates the need for detailed information about rates in the alignment provided by AIR.

The great need for efficient bioinformatic tools in reconstructing multi-gene alignments for phylogenomic inferences has over the last years been met by several new applications, such as Concatenator, IDEA, SCaFoS, IDEA and ASAP [36-40]. Several of these have overlapping functionalities with the AIR package, but the AIR is unique in combining key steps for constructing multi-gene alignments and evolutionary rate estimations. Most importantly AIR allows trimming of alignments according to the evolutionary rates and the users' preferences. Site rates estimation can be based on multiple phylogenies that account for uncertainties in the phylogeny. Several different criterions can be used for removing sites, either based on rate categories or site rates, which reduces the possibility of removing too many or few sites from the alignment. Monitoring of the site removal process is easy by using the colored alignment output files from the AIR.

In contrast to the vast majority of other programs, the AIR package is easily accessible on the web and does not require cumbersome installation on local computers. AIR is implemented on the Bioportal where users have their own file directories and can access several widely used programs in molecular evolution and ecology. The result files from the AIR programs can also be easily downloaded and applied in downstream analyses at other web-based bioinformatics services (such as  and ). This makes the AIR package user-friendly and efficient. As AIR will process files on a large computer cluster, with the prospect of being linked to a larger grid infrastructure in future, there is currently no restriction on the size of the input sequences.

## Availability and requirements

Project name: AIR version 1.1

Project home page: 

Operating system(s): Platform independent

Programming language: SQL, Perl, Python and PHP

Other requirements: Apache webserver

License: GNU - GPL

Any restrictions to use by non-academics: AIR-Identifier uses PAML with license for academic use. Non-academic users still can use AIR-Appender and AIR-Remover at . Test dataset for all programs of AIR is available at .

## Authors' contributions

SK conducted the programming of AIR-Appender, AIR-Identifier and AIR-remover, wrote the paper and implemented the applications on the Bioportal. ÅS contributed with programming of AIR-Appender. RO and FB tested the AIR programs and contributed with writing of the manuscript. PE contributed with programming and implementation of the AIR on the Bioportal. ÅS, PE, TR, BHM and AB programmed the Bioportal. KST funded and designed the project, supervised the process, wrote the first draft of the AIR paper. KST and AB initiated the Bioportal service, and KST is leading the development of the service. All authors read and approved the final manuscript.
